# Calculation of Tajima’s *D* and other neutrality test statistics from low depth next-generation sequencing data

**DOI:** 10.1186/1471-2105-14-289

**Published:** 2013-10-02

**Authors:** Thorfinn Sand Korneliussen, Ida Moltke, Anders Albrechtsen, Rasmus Nielsen

**Affiliations:** 1 Centre for GeoGenetics, Natural History Museum of Denmark, University of Copenhagen, Oestervoldgade 5-7, DK-1350, Copenhagen, Denmark; 2 Department of Human Genetics, University of Chicago, 920 E. 58th Street, CLSC 5th floor, Chicago, IL 60637, USA; 3 The Bioinformatics Centre, Department of Biology, University of Copenhagen, Ole Maaloes Vej 5, DK-2200, Copenhagen, Denmark; 4 Departments of Integrative Biology and Statistics, UC-Berkeley, 4098 VLSB, Berkeley, California 94720, USA

**Keywords:** Next-generation sequencing, Darwinian selection, Neutrality tests

## Abstract

**Background:**

A number of different statistics are used for detecting natural selection using DNA sequencing data, including statistics that are summaries of the frequency spectrum, such as Tajima’s *D*. These statistics are now often being applied in the analysis of Next Generation Sequencing (NGS) data. However, estimates of frequency spectra from NGS data are strongly affected by low sequencing coverage; the inherent technology dependent variation in sequencing depth causes systematic differences in the value of the statistic among genomic regions.

**Results:**

We have developed an approach that accommodates the uncertainty of the data when calculating site frequency based neutrality test statistics. A salient feature of this approach is that it implicitly solves the problems of varying sequencing depth, missing data and avoids the need to infer variable sites for the analysis and thereby avoids ascertainment problems introduced by a SNP discovery process.

**Conclusion:**

Using an empirical Bayes approach for fast computations, we show that this method produces results for low-coverage NGS data comparable to those achieved when the genotypes are known without uncertainty. We also validate the method in an analysis of data from the 1000 genomes project. The method is implemented in a fast framework which enables researchers to perform these neutrality tests on a genome-wide scale.

## Background

In the past decade there has been considerable interest in detecting natural selection in humans and other organisms [1-4] from DNA sequence data. An often used approach for detecting selection is to use a neutrality test statistic based on allele frequencies, with Tajima’s *D* being the most famous. Such frequency based tests have been used to identify a number of genes, that have undergone selection: lactase [5], ABO blood group [6], the HLA immune complex [7], and the calcium pathway trpv6 [8].

In recent years Next-Generation Sequencing (NGS) has revolutionized the field of genetics in general and population genetics in particular. The underlying technology behind these different high-throughput sequencers are different [9,10], but common is the unprecedented level of data produced, allowing population geneticists access to full genome data for large population samples at an affordable price.

Applying the neutrality tests directly to the genotypes obtained from SNP chips can lead to biased results because of the way the SNPs were selected. Even though work has been done to correct for this [11], corrections are not possible without knowledge of the selection criteria used to ascertain SNPs for the specific chip. NGS data do not suffer from such ascertainment biases. However, with low and medium depth (<20×), genotype calling from NGS data can lead to other biases [12]. The biases will depend on the method used for genotype calling. Early genotype calling methods were based on simple criteria, such as calling a heterozygous site, if the proportion of non-reference alleles is between 20% and 80% [13,14]. Most commonly used genotype callers today are probabilistic, i.e. they are based on genotype likelihoods (GLs) [15]. A GL is the probability of the observed sequencing data given a genotype. GLs are obtained from the raw NGS read data, typically using information such as position in the read, quality score, and possibly type of mutation [16,17]. A standard approach for data analysis is to call genotypes, and then estimate neutrality statistics. Most genotype callers are relatively conservative and only call a site to be heterozygous if there is substantial evidence that it actually is heterozygous. Such methods will tend to underestimate the allele frequency of the minor allele. Less conservative methods will tend to produce too many rare variants. In either case, an unbiased estimate of the allele frequency cannot be obtained for all classes of allele frequencies [1,18,19]. In some studies, an attempt to alleviate this problem has made by only calling a genotype when there is substantial statistical evidence supporting the genotype call. Such approaches will generate a considerable amount of missing data which leads to biases if not adequately dealt with [20]. The effect of these errors on downstream analyses can be severe. For most data produced, the strongest bias has been an excess of singletons (derived alleles segregating in a single copy in the sample). If just a single individual in a sample is miscalled to be heterozygous in a monomorphic site, the site will appear as polymorphic singleton site. The effects of genotype calling for downstream population genetic analyses have been extensively illustrated using simulations by [12]. Other approaches have been suggested: Achaz, 2008 [21] proposed modified statistics that excludes the singletons to address the problem of high error rate in sequencing data. Liu et al., 2010 [22] does not use the quality scores but uses an error rate for each nucleotide for all samples and [23] which uses a fixed error rate. This is contrasted by the methods of [19,24] that can use quality scores for SNP discovery and incorporating the quality scores into the parameter estimation directly.

We will here show, using both simulated and real human data that, that Tajima’s *D* calculated using genotypes called from NGS data can lead to severely biased results. The level of bias depends on the sequencing depths and error rates, but disturbingly it also depends on whether or not the data set is neutral or not. This in effect leads to lower power to detect selection.

We argue, that the solution to this problem is to avoid genotype calling, but instead incorporate uncertainty in the genotypes through direct analyses of the genotype likelihood (GL). This approach also solves the issue of missing data, and the problem of inferring which sites are variable. We propose two methods for estimating the neutrality tests statistics from low depth NGS data. In both methods the estimates are based on GLs instead of called genotypes. The first approach is based on obtaining a Maximum Likelihood (ML) estimate of the sample allele frequency spectrum. This approach takes all the uncertainty of the NGS data into account, and provides estimates of the neutrality tests statistics from the estimated sample frequency spectrum. However, it is too slow for genome-wide window based scans. The second approach is computationally feasible for entire genomes of any magnitude. It uses an empirical Bayes approach, that also uses an ML estimate of the site frequency spectrum. This estimate is used as a prior for calculating site specific posterior probabilities for the sample frequency spectrum. We show, using both simulated and real low depth NGS data, that the test statistics estimated using the empirical Bayes approach are at most weakly biased and provide a computationally attractive alternative to genotype calling methods.

## Methods

### Estimators of *θ* and neutrality tests

In a set of *n* aligned DNA sequences, the frequency spectrum (SFS) is defined as the vector *η* = (*η*_*k*_)_*k* = 0,…,*n*_ where *η*_*k*_ is the number of sites with *k* derived alleles. A derived allele is a new mutation and the ancestral allele is the nucleotide occurring in the site before mutation. Notice that these population genetic models assume an infinite sites model, and there is therefore an unambiguous definition of ancestral and derived. In real data, outgroup information is used to estimate which allele is ancestral and which allele is derived. Using this notation, the number of segregating, i.e. polymorphic, sites is then given by $S=\sum_{i=1}^{n-1}\eta_{i}$. The most commonly applied estimators of *θ*=4 *Nμ*, (*N* is the effective population size and *μ* the mutation rate) are linear functions of the frequency spectrum. These estimators take the general form: $\hat{\theta }=\sum_{i=0}^{n}\alpha_{i}\eta_{i}$, where *α*_*i*_ are different vectors of weights used in constructing the estimators. Some of the common estimators include:

• ${\hat{\theta }}_{w}{=a}_{1}^{‒1}\sum_{i=1}^{n-1}\eta_{i},\;$ with $a_{1}=\sum_{i=1}^{n-1}i.$[25]

• ${\hat{\theta }}_{\pi }={\left(\begin{array}{c} n\\ 2 \end{array}\right)}^{-1}\sum_{i=1}^{n-1}i\left(n-i\right)\eta_{i}.$[26]

• ${\hat{\theta }}_{\mathrm{FL}}=\eta_{1}.$[27]

• ${\hat{\theta }}_{H}={\left(\begin{array}{c} n\\ 2 \end{array}\right)}^{-1}\sum_{i=1}^{n-1}i^{2}\eta_{i}.$[28]

For more background on these estimators, see for example [29]. In brief, notice that the estimators differ in how they weight polymorphisms with different allele frequencies. The ${\hat{\theta }}_{w}$, and ${\hat{\theta }}_{\pi }$ estimators are symmetric in that they assign equal weight to ancestral and derived alleles of the same frequency. However, ${\hat{\theta }}_{\pi }$ assigns more weight to alleles segregating at intermediate frequencies while ${\hat{\theta }}_{w}$ weights all categories equal. In contrast, ${\hat{\theta }}_{\mathrm{FL}}$ only assigns positive weight to derived singletons and ${\hat{\theta }}_{H}$ assigns more weight to derived alleles of high frequency. The first two estimators can be calculated without knowledge of the ancestral by using the folded SFS, $\eta^{*}={\left(\eta_{k}^{*}\right)}_{k=0,\ldots ,n/2},\eta_{k}^{*}=\eta_{k}+\eta_{n-k}$, assuming *n* is even. The latter two estimators require knowledge regarding ancestral states, i.e. they use the unfolded SFS (*η*).

None of these estimators are maximum likelihood estimators (except in the trivial case of *n* = 2). However, they are commonly used to construct tests statistics used to test if a predicted SFS fits an observed SFS. In a standard neutral model *E*[*η*_*i*_] = *θ*/*i*[26]. Selection, as well as other violations of the model assumptions such as population structure or population growth, will cause deviations from this expectation [30]. The SFS based neutrality tests capture this effect by the use of test statistics that are constructed as the standardized difference between two different *θ* estimators. They have the general form:

$$T=\frac{\theta_{1}‒\theta_{2}}{\sqrt{\mathrm{var}\left(\theta_{1}‒\theta_{2}\right)}},$$

where the variance usually is the variance expected under a standard neutral model without recombination. For example, Tajima’s *D* is given by setting ${\hat{\theta }}_{1}={\hat{\theta }}_{\pi }$ and ${\hat{\theta }}_{2}={\hat{\theta }}_{w}$. Tajima’s *D* is often used for detecting selective sweeps, i.e. the effect of an advantageous mutation going to fixation in the population. After fixation, of an advantageous allele, there is an excess of rare alleles in the population. This will cause Tajima’s *D* to become negative as the expectation of ${\hat{\theta }}_{\pi }$ will be smaller than the expectation of ${\hat{\theta }}_{w}$, when the SFS contains relatively more rare alleles than expected under a standard neutral model.

### Genotype likelihoods

The standard method for representing uncertainty in NGS data is in terms of Genotype Likelihoods (GLs). All methods discussed in this paper are based on such GLs. We, therefore, first introduce the GL calculations before describing the methods used for calculating neutrality statistics. The GL for an individual in a particular site is defined as the probability of the observed read data (*D*) in the site given the genotype type (*G*) of the individual in that site:

$$\begin{array}{l} L\left(G=\left\{A_{1},A_{2}\right\}\;|D\right)\propto \mathrm{Pr}\left(D|G=\left\{A_{1},A_{2}\right\}\right)\;,\\ \;A_{1},A_{2}\in \left\{A,C,G,T\right\}. \end{array}$$

The GL can be calculated by assuming independence of the reads covering a position. Some methods take into account the position within the read and does recalibration which take into account cycle dependencies [17]. The implementation in [17] however assumes that all reads have the same length. The widely used SAMtools uses a model derived from the Maq program [15,31]. These GL methods are all single sample based in contrast to the method of [32], which estimates type specific errors for multi samples jointly. In this paper we base our simulations on a simplified method of [16].

### Model

#### Full maximum likelihood

Several recent papers have suggested methods for estimating the SFS from NGS data [31,33-35]. One approach that we will explore for calculating neutrality statistics (*T*) for a region, is to estimate the SFS, *η* = (*η*_*k*_)_*k* = 0,…,*n*_, using one of these methods, in this case the maximum likelihood method by [35] (${\hat{\eta }}_{\mathrm{ML}}$), and then calculate *T* using estimated values of η instead of the (unknown) true values. As (${\hat{\eta }}_{\mathrm{ML}}$) has previously been shown to perform well under simple conditions, we would expect this approach for calculating the frequency spectrum to perform well under similar conditions. We note that the uncertainty regarding genotypes and the effect of sequencing and mapping errors are incorporated in this approach through the calculation of GLs as described in the section above, but we will otherwise refer readers to [35] for information regarding algorithmic details. Neutrality tests are normally performed using sliding windows in a genome. Maximum likelihood estimation of the SFS for all windows may be computationally challenging. For this reason we also explore other approaches below.

#### Empirical Bayes

In a second approach we instead calculate the posterior probability of the allele frequencies for each site, and combine them into a joint estimate of the SFS as in [33], i.e. for each site we calculate:

$$\mathrm{Pr}\left(x=j|D\right)=\frac{\mathrm{Pr}\left(D|x=j\right)\mathrm{Pr}\left(x=j\right)}{\sum_{i=0}^{2n}\mathrm{Pr}\left(D|x=i\right)\mathrm{Pr}\left(x=i\right)},$$

where *x* is the sample allele frequency in that site, and *n* being the number of diploid samples. Pr(*D* | *x* = *j*) can be calculated fast using a dynamic programming algorithm described in detail in references [33,35], and we refer readers to these publications for details. The *j*th category of the SFS, is then estimated by summing Pr(*D* | *x* = *j*) over all sites in the region. The prior, Pr(*x* = *i*) can be defined, for example from a ML estimate of the SFS from the entire genome, or from a reasonable subset of the genome. The computational advantage of this approach is that we avoid optimization of all windows of the genome. We consider this an empirical Bayes approach as the prior is estimated from the data. Another advantage of this approach is that the estimates of *θ* calculated are actual posterior expectations of *θ*. This is true because all are linear functions of the *η*_*i*_ ’s, i.e.

$$E\left(\hat{\theta }|D\right)=\sum_{i=0}^{n}\alpha_{i}E\left[\eta_{i}|D\right].$$

#### Calling genotypes

To assess the performance of our method we will compare with a more simple method that estimates the SFS by calling genotypes for each individual using the same genotype likelihood calculations as used to estimate the SFS in the previous approaches. We evaluate two commonly used GL-based calling procedures, that both assume that the major and minor allele are defined:

$$\begin{array}{l} G_{\mathrm{mLike}}=\underset{g\in \left\{0,1,2\right\}}{\arg\max}\left\{\mathrm{Pr}\left(D|G=g\right)\right\},\\ G_{\mathrm{hwe}}=\underset{g\in \left\{0,1,2\right\}}{\arg\max}\left\{\left(\begin{array}{c} 2\\ g \end{array}\right){\hat{f}}^{g}{\left(1-\hat{f}\right)}^{2-g}\mathrm{Pr}\left(D|G=g\right)\right\}. \end{array}$$

Notice that we here, for notational convenience, code genotypes as elements of {0, 1, 2} instead of pairs of nucleotides that each are elements of {A, C, T, G}. The first method chooses the genotype with the highest GL. The second procedure (a type of maximum *a posteriori* probability estimate) calls genotypes by first estimating the sample allele frequency *f* from a larger group of individuals, and then uses this estimate of *f* as a prior on the 3 genotypes using the assumption of Hardy-Weinberg equilibrium (HWE).

Simply calling all genotypes would result in too many false heterozygotes due to the sequencing errors, resulting in too many variable sites and an inflation of the singleton category of the SFS. Therefore, we also need to select an inclusion criterion for the sites we deem variable, i.e. sites in which we allow the existence of heterozygotes (SNP calling). To do so we first perform a likelihood ratio test of the null hypothesis H_0_: *f* = 0, combining the data from all individuals [32]. We do so assuming that all sites are di-allelic, and use the approach described in the supplementary of [36] to identify the two most likely alleles for a given site. Throughout we use the maximum likelihood estimator of *f* by [32].

#### Simulations

To assess the performance of our estimators we simulate genomic data under a standard neutral model and under models that deviate from the neutral model using msms [37]. We set the population size, mutation rate and the recombination rate to realistic values for humans, *N* = 10, 000, *μ* = 2.35 · 10^− 8^, *r* = 10^− 8^[38,39] and use an infinite sites model. As msms only prints out variable sites with binary coding, we insert invariable sites in the sequences and convert from binary coding to nucleotides by sampling randomly with equal probability from all four nucleotides. We then collapse the simulated haplotypes into genotypes. For the non-neutral scenarios we simulate strong positive selection under an additive model with a selection coefficient of 0.1.

To simulate NGS data based on the msms genotypes, we use a model similar to the model assumed for GL calculations in GATK [40]. The simplified GATK model uses only base quality information and calculates the GL for a single site as:

$$\begin{array}{l} \mathrm{Pr}\left(D|G=\left\{A_{1},A_{2}\right\}\right)=\prod_{i=1}^{M}\mathrm{Pr}\left(b_{i}|G=\left\{A_{1},A_{2}\right\}\right)\\ \;=\prod_{i=1}^{M}\left(\frac{1}{2}p\left(b_{i}|A_{1}\right)+\frac{1}{2}p\left(b_{i}|A_{2}\right)\right),\\ p\left(b|A\right)=\left\{\begin{array}{cc} \frac{e}{3} & b\neq A\\ 1-e & b=A. \end{array}\right) \end{array}$$

where *M* is the sequencing depth, *b*_*i*_ the observed base in read *i*, *e* is the probability of error and *G* = {*A*_1_,*A*_2_}. In our simulations, we assume an equal error rate for all bases and for all sites. The sequencing depth is sampled from a Poisson distribution with mean equal to the specified mean depth. The simulations then proceed by first simulating *G* using msms and then simulating *D* in accordance with the formula given above. The latter stage is achieved by sampling *M* nucleotides from *G*, and then introducing errors independently in each of them with probability *e*. For each site GLs are then calculated according to the GATK model given above.

It should be clarified that we do not actually simulate reads, but sample bases for every site independently. Hence there is a dependency in sequencing depth between adjacent sites in real data that we do not model.

#### HapMap 2 and 1000genomes

For evaluating the performance of the estimators on real data we used 15 unrelated CEU samples from HapMap phase 2 data [41], sequenced by the 1000 genomes project [42] using Illumina sequencing. Based on the mapped reads we used ANGSD http://www.popgen.dk/angsd to align the 15 mapped samples and calculate the genotype likelihoods using the GATK error model. Ancestral states for all sites were obtained from the multiz46way dataset http://hgdownload.cse.ucsc.edu/goldenPath/hg19/multiz46way/ available from the UCSC browser.

## Results

### The effect of genotype calling for low or medium coverage data

**Figure 1:**
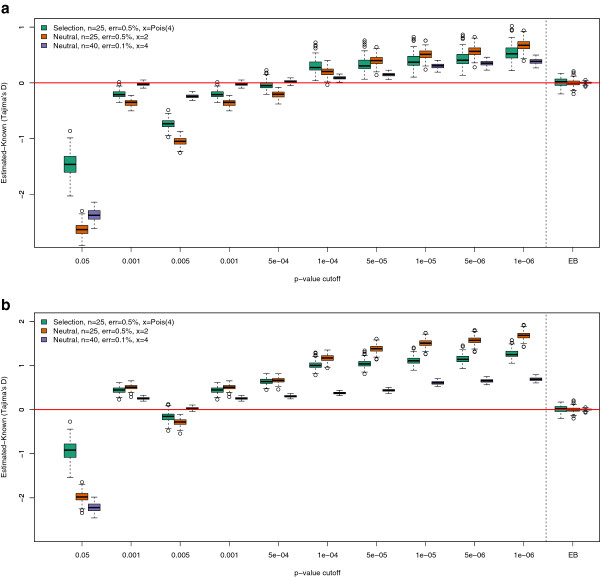
**The effect of genotype calling for low or medium coverage data using Tajima’s *D*.** The difference between estimated and known Tajima’s *D* statistic for three different scenarios with 10 different p-value cutoffs. Each box is estimated on the basis of 100 1 MB regions. Subfigure ‘**a)**’ is based on genotypes called using the frequency as prior, GC-hwe, and Subfigure ‘**b)**’ is based on genotypes called using a maximum likelihood approach, GC-mLike. We have included the EB method for the 3 different scenarios in the right side of the figures. Notice that no single best cutoff can be chosen across the three different scenarios for the genotype calling based methods.

In order to evaluate the performance of the estimators we simulated multiple genomic regions both without selection and with strong positive selection. We first attempted to identify an optimal p-value cutoff to use in the likelihood ratio tests applied in the genotype calling methods. Figure 1 depicts the distribution of the difference between the estimated and known value of Tajima’s *D* for 10 different p-value cutoffs, along with the results using our EB approach. Each box represents the estimate from 100 1 MB regions. We simulated two neutral scenarios (25 and 40 samples) and one scenario with strong positive selection (25 samples) and we used a mean depth of 2 and 4 with an error rate of 0.5% and 0.1%. For the scenario with selection we used an error rate of 0.5%, but sampled the mean sequencing depth between the different samples from a Poisson distribution with mean of 4. For the same three scenarios we also estimated Fu and Li’s *D* and *F* statistics [27] (Additional file 1: Figure S1 and Additional file 2: Figure S2). We see that it is not possible to choose a single p-value cutoff that is unbiased for all the examined scenarios (Figure 1, Additional file 1: Figure S1 and Additional file 2: Figure S2). Any particular cut-off for genotype calling will result in biases in the estimate of Tajima’s *D* that will depend on the true value of Tajima’s *D*, error rates, and sequencing depth. These results suggest that, for low or medium coverage data, using called genotypes is in general problematic no matter what cutoff is used and will give rise to biased results that are context dependent.

### The effect of SNP calling criteria when calling genotypes

**Figure 2:**
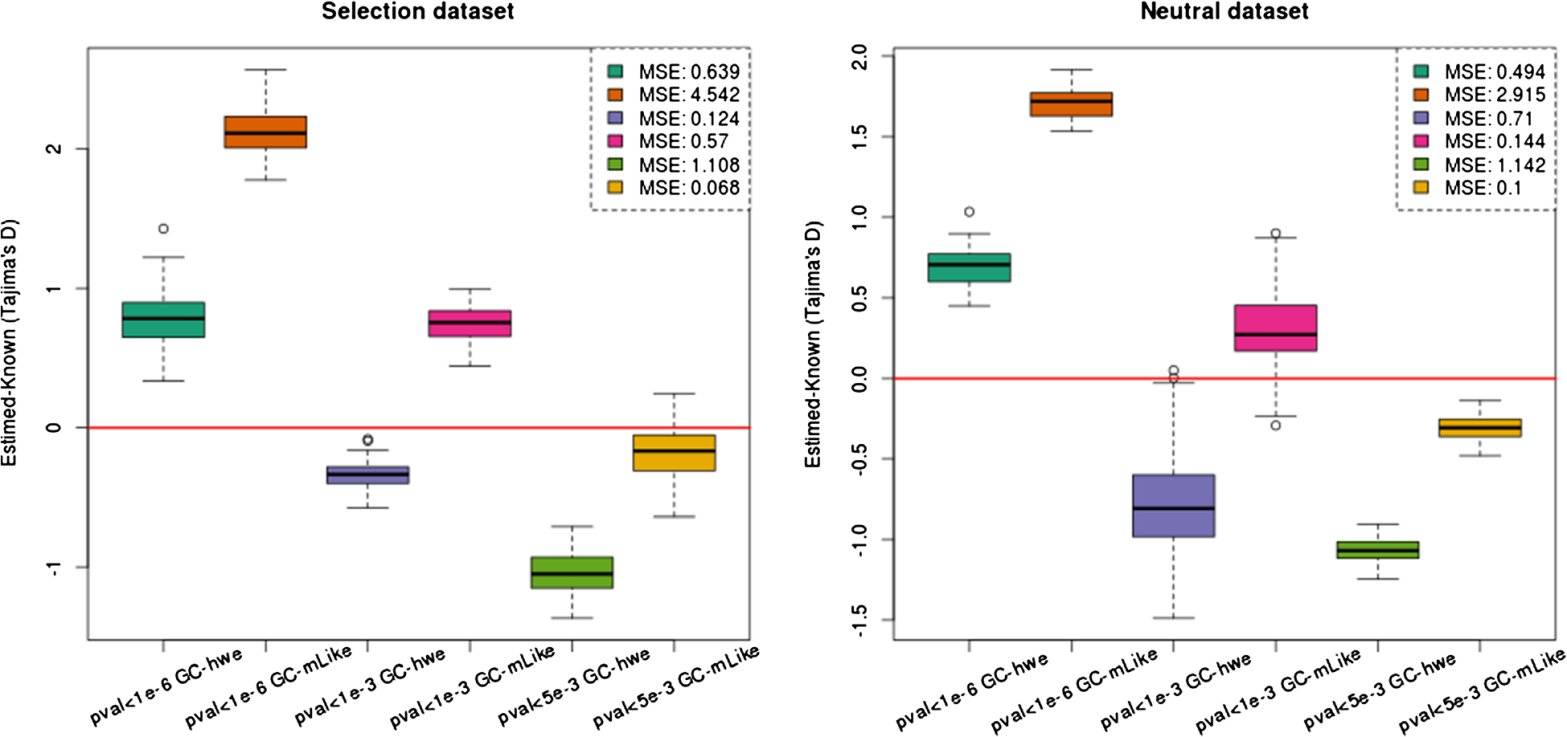
**The effect of SNP calling criteria on the variance when calling genotypes.** Comparison of the difference between our estimated Tajima’s *D* and the known Tajima’s *D*. These plots are based on a scenario with depth 2× and error rate 0.5% and show the difference of different p-values used for the LRT test. Left figure is the selection dataset and right figure is the neutral dataset. Notice that the 10^-6^ cutoff has quite the same variance in both plots. We observe that the 10^-3^ cutoff has less variance on the selection dataset, but more variance in the neutral dataset. We see the opposite correlation with regards to the 5 × 10^-5^ cutoff.

To further examine to what extent the bias varies according to the p-value cutoff used in the LRT test for inferring variable sites, we summarized the distribution of estimated and known values in boxplots (Figure 2). This was done for both genotype calling methods and for three different critical values (10^-6^, 10^-3^, 5 × 10^-3^). There are some obvious biases due to SNP calling. Less conservative SNP calling will cause an excess of (false) low frequency variants, and therefore an underestimation of Tajima’s *D*, and more conservative SNP calling will cause a deficiency of rare alleles and, thereby, overestimate Tajima’s *D*. Also notice that for both genotype calling methods, the p-value threshold of 10^-3^ has less variance in the selection datasets compared to the neutral datasets, whereas the opposite trend is true with the more relaxed threshold of 5 × 10^-3^ (Figure 2).

In the following sections, we therefore compare our methods to results using several different cutoffs for genotype calling.

### Estimating Tajima’s *D* using ML estimates of the SFS

**Figure 3:**
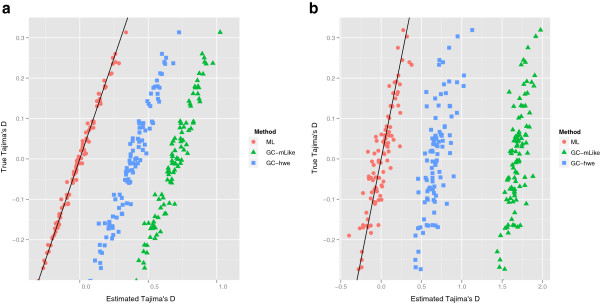
**Estimating Tajima’s *D *using ML estimates of the SFS.** These two plots are based on neutral sets of scenario, each plotted data point is an estimate of Tajima’s *D* for a 1 Mb region. Subfigure ‘**a)**’ is high depth (8×), with an error rate of 1.0% and using a p-value of 10^-6^ and subfigure ‘**b)**’ is low depth (2×) with an error rate of 0.5%. For our full ML method in the high depth scenario all values fall in the vicinity of the y = x line, but shows higher variance for the low depth as expected. Notice that for the stringent cutoff both genotype calling methods overestimates.

We next simulated two neutral scenarios consisting of 25 samples and simulated 100 simulations of 1 MB regions for each. For the first neutral scenario we simulated fairly high sequencing depth and assumed a high error rate (8×, 1%). For the second neutral scenario we simulated a lower depth and lower error rates (2×, 0.5%). We inferred Tajima’s *D* values from the simulated haplotypes, which we will here denote as the true values. Values of Tajima’s *D* were then estimated from the simulated sequencing data using our full ML approach and from genotypes called from the sequencing data (Figure 3). For called genotypes we only included sites that were likely to be polymorphic with a p-value less than 10^-6^. In both scenarios our approach gives very similar results to the estimates from the true haplotypes while the approaches based on genotype calling shows large biases as expected from the results presented in the previous section. Not surprisingly, we observe the least accurate results for the low depth scenario, but even at 8X coverage there are substantial biases.

### The effect of sample size and sequencing depth

**Figure 4:**
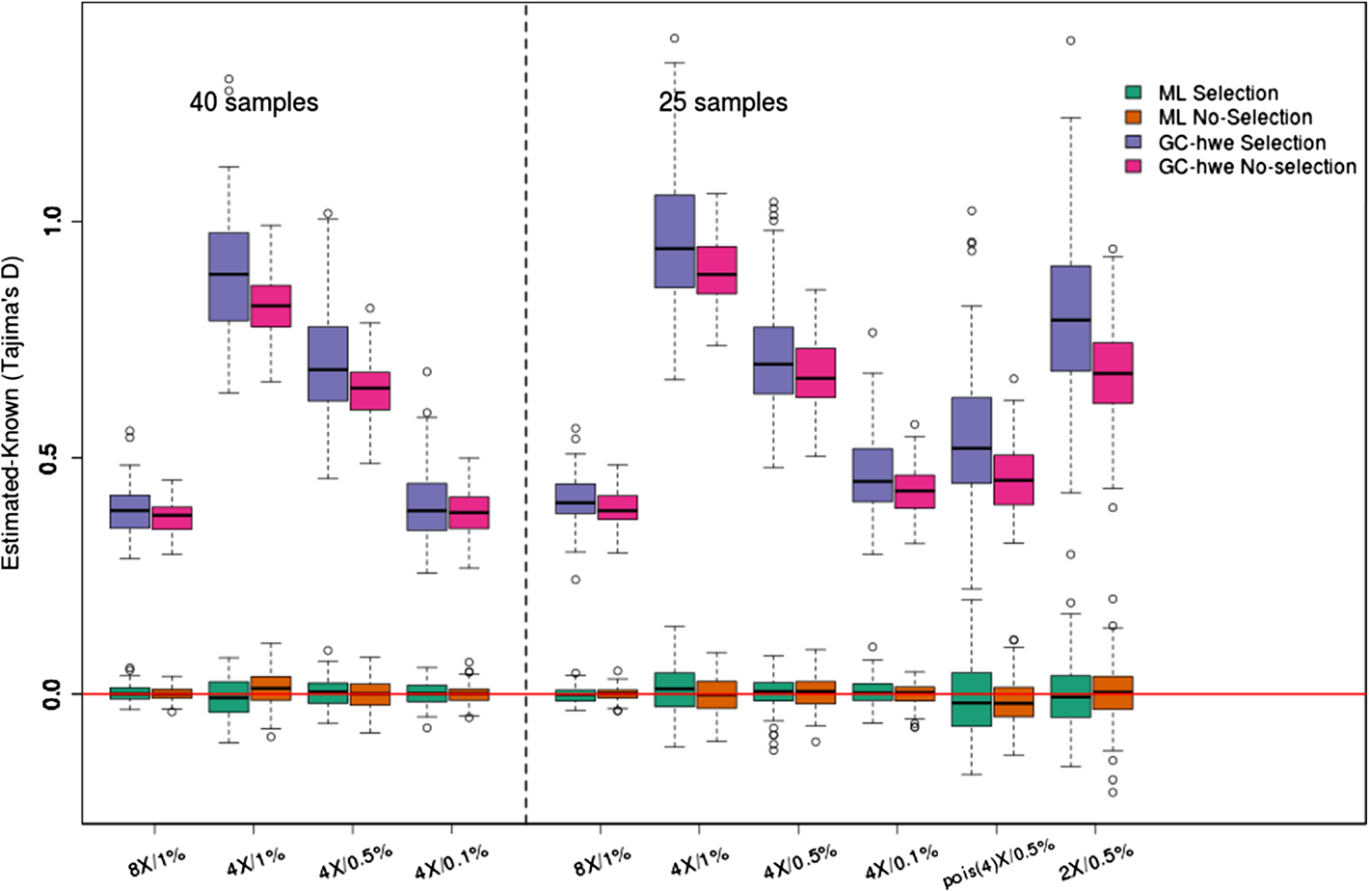
**The effect of sample size and sequencing depth.** Boxplot of our estimated values minus the known value. The green/orange boxes indicate our full maximum likelihood method, whereas the other boxes are the genotype calling methods. We have generated 10 scenarios with and without selection, therefore each box represents different scenarios each with 100 data points estimated on the basis of the 100 × 1 Mb datasets. For these analysis we used a p-value of 10^-6^. Notice that the full ML method is mostly unbiased, but the variance is affected by the error rate and sequencing depth as expected. The genotype calling shows large biases that depend on sequencing depth, error rate and whether or not the region is under selection or not.

The effect of sequencing depth and error rate is further examined in Figure 4. Here we show the distribution of the differences between the true and the estimated Tajima’s *D* values. As above, for each scenario we perform 100 simulations of 1 MB regions. The first 4 sets of simulations are conducted with sample sizes of *n* = 40, and the last set of 6 simulations are conducted with sample sizes of *n* = 25. As can be seen, large error rates and sequencing depth affects the variance of the estimate for the full maximum likelihood method but the estimates remain approximately unbiased. In contrast, the genotype calling (GC) approaches show large biases that depend on sequencing depths and error rates. Similar results are observed for both sample sizes. The mean simulated value (across the 100 repetitions) for three of the estimators of *θ* are shown in Table 1. The full likelihood method is mostly unbiased whereas the GC based methods are biased to a degree that depends on whether the region is under selection or not.

**Table 1:** Mean estimate of theta estimators for 100x1MB regions for 25 samples

	** Selection**		**No selection**			
**Method**	***θ***_***w***_	***θ***_***π***_	***θ***_***FL***_	***θ***_***w***_	***θ***_***π***_	***θ***_***FL***_
Known	649.8	567.1	745.1	930.0	923.5	939.0
ML	648.6	567.9	734.7	932.4	923.7	968.3
GC-hwe 10^-6^	341.7	372.1	72.2	541.3	640.3	99.9
GC-mLike 10^-6^	341.7	497.1	32.9	541.3	768.7	44.8
GC-hwe 10^-3^	556.1	434.7	583.5	784.8	712.4	680.6
GC-mLike 10^-3^	556.1	598.9	360.4	784.8	897.9	410.1
GC-hwe 10^-5^	1011.0	594.8	653.7	1244.4	874.1	752.8
GC-mLike 10^-5^	1011.0	831.8	420.4	1244.4	1131.6	471.8

The genotype likelihoods are simulated with an error rate of 0.5% and an average depth of 2 ×.

### Application to data simulated with a selective sweep

**Figure 5:**
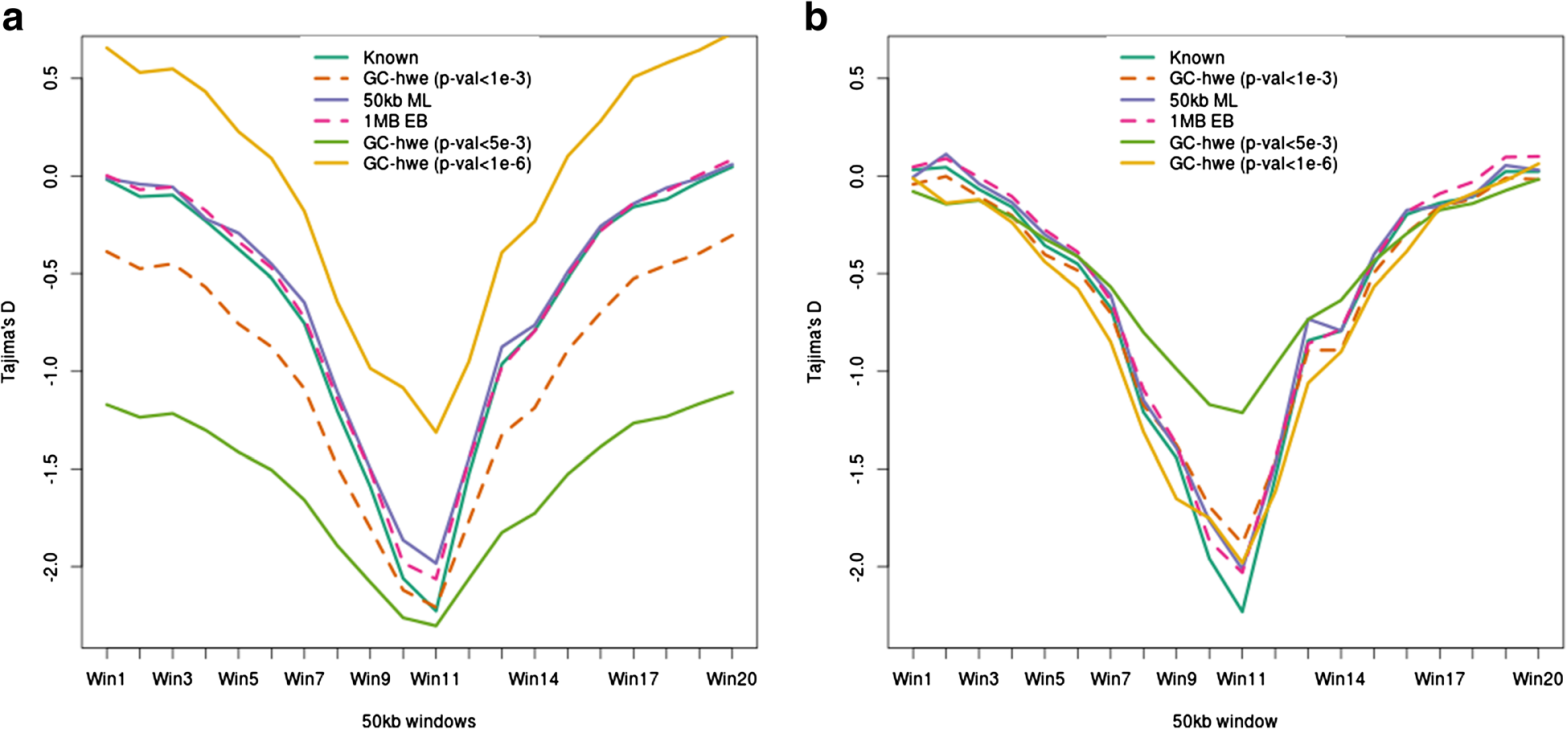
**Difference between our estimators under a selective sweep.** Mean value for our estimated Tajima’s *D*, for every 50 kb windows for 100 1 MB region for 25 samples. For the most progressive LRT cutoff some windows did not have data. This plot is based on a depth of 2× and an error rate of 0.5%. Figure **a)** is using the raw Tajima’s *D* estimate for the genotype calling methods. In Figure **b)** we have standardized the genotype calling methods relative to the estimates from a dataset of 100 1 MB neutrally evolving regions.

In typical applications to genome-wide data, Tajima’s *D* will usually be calculated separately for multiple smaller regions, often in a sliding window. We therefore separated the 1 MB regions into 20 subregions each of 50 kb. We used both the full maximum likelihood method for each subregion and applied the empirical Bayes (EB) method. The full maximum likelihood estimate from the whole 1 Mb region was used as a prior for the EB method. All of the methods show a decrease in Tajima’s *D* values around the site under selection (Figure 5). However, the threshold for inferring polymorphic sites has a strong influence on the performance of the GC methods. Note that because the full likelihood and the EB method do not introduce a bias in the estimate of Tajima’s *D*, they do not have to be standardized using a neutral data set if the underlying demographic model is known.

**Figure 6:**
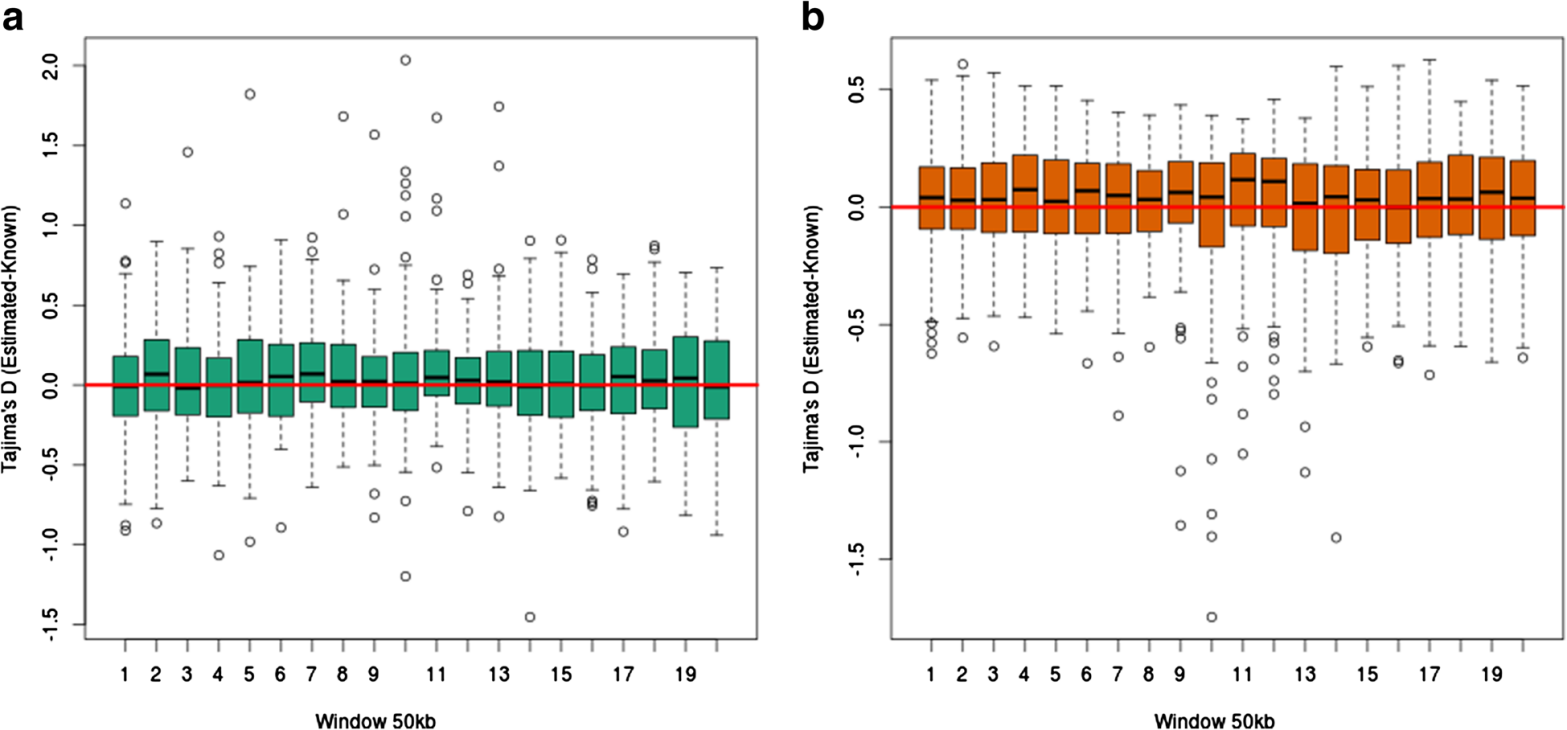
**Difference between the full ML and the EB method under a selective sweep.** Difference between estimated Tajima’s *D* and known Tajima’s *D*, left plot ‘**a)**’ is using the ML for every 50 kb region, right figure ‘**b)**’ is using the EB approach with a 1 Mb estimated SFS as prior for all 50 kb regions. The targeted site for selection is in the middle and we see that the local 50 kb ML has some very positive outliers in this region. This is contrasted by the EB method where we have some negative outliers. However we can see that the median for the EB method in the middle region is shifted to a positive value, whereas the median for the 50 kb ML is overall relatively close to zero. Also notice the difference of scaling in the y-axis.

In Figure 6 we illustrate the distribution of the difference between the estimated Tajima’s *D* and the true value for every window for the ML estimator and the EB estimator. We note that the variance is larger for the full ML approach than the EB approach. This is further examined in Additional file 3: Figure S3 where we have plotted the difference in Mean Squared Error (MSE) for the same 20 subregions with the ML method and the EB method. We notice that the MSE for the EB method is smaller.

### Effect of the prior

**Figure 7:**
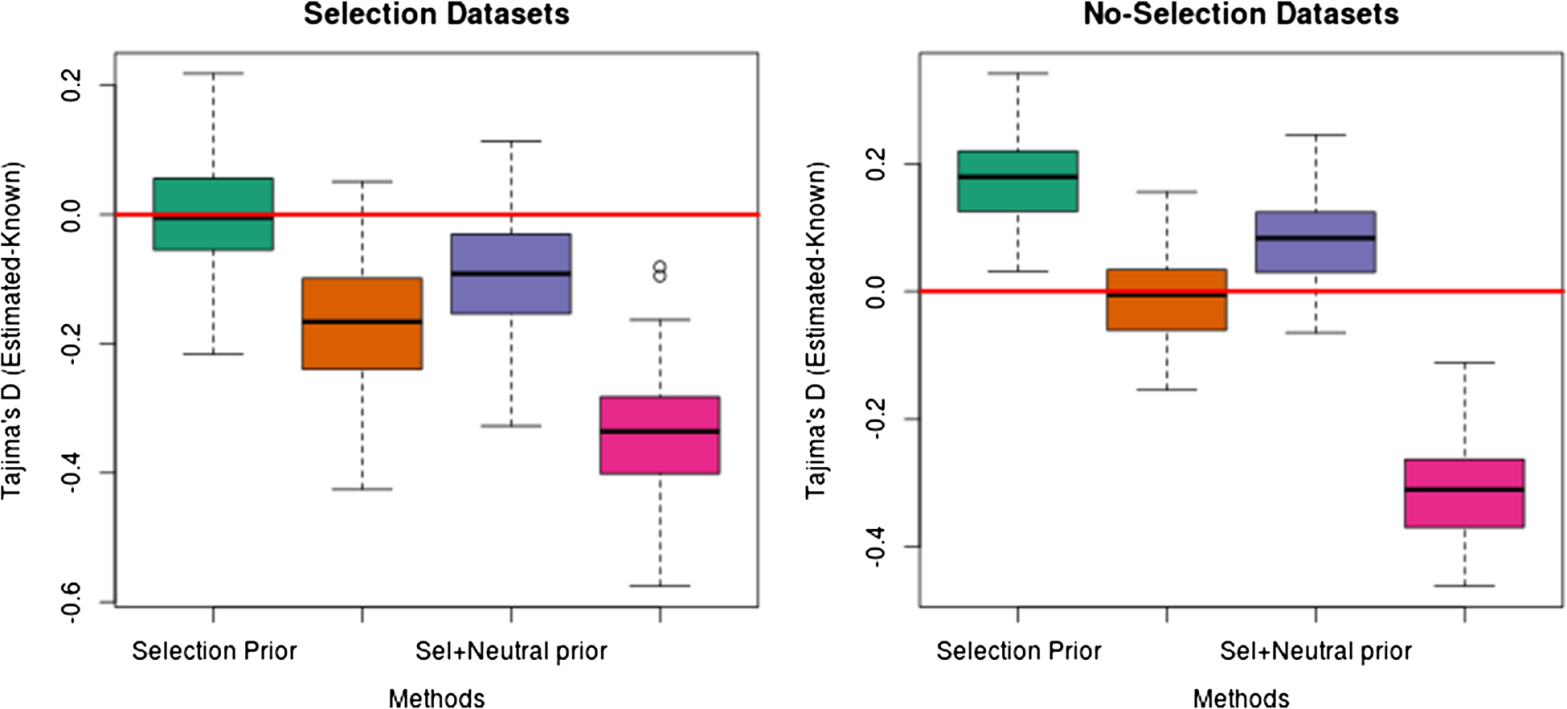
**Effect of different priors for the EB method using the Tajima’s *D *test statistic.** Boxplots for the difference between our estimate of Tajima’s *D* and the known value, the orange box is the neutral genome-wide prior. These plots are based on a depth of 2× and an error rate of 0.5%.

For the EB method, the prior can have an impact on the estimates of Tajima’s *D*. In Figure 7 we explore the effect of the prior on the estimated values. We use three different priors. 1) a prior estimated on a neutral data set of 100 MB, 2) a prior estimated for a 100 MB region under selection 3) a prior based on both types of regions in equal proportion. As can be seen in Figure 7 when applying the neutral prior to the neutral datasets or the selection prior on the selection dataset the estimates are approximately unbiased. However, applying the “wrong prior” tends to either underestimate or overestimate the selection signal. Perhaps a bit surprisingly, using the neutral prior on the selection datasets tends to overestimate the signal, while using the selection prior on the neutral datasets tends to underestimate it. This is explained by the fact that a region of selection will have less variability than a neutral region. A prior trained on a neutral dataset will therefore have a higher level of variability than the true level of variability in a region of selection. Applying a neutral prior will therefore allow more variability, and due to sequencing errors these sites will mostly be singletons. This excess of singletons will have the largest impact on ${\hat{\theta }}_{w}$, and we will therefore underestimate Tajima’s *D*. The opposite argument explains why the selection prior will overestimate Tajima’s *D* when applied to a neutral dataset.

We observe the same trend for other neutrality tests statistics that can be seen in Additional file 4: Figure S4 and Additional file 5: Figure S5. However, the other investigated neutrality test statistics, Fu & Li’s *D* and *F*, are not estimated with the same accuracy as Tajima’s *D*. For both selection and neutral data sets, we seem to underestimate the statistics with the EB approach and neutral prior (Additional file 4: Figure S4 and Additional file 5: Figure S5). From figure (S4,S5) we also see that we have problems estimating the true value in the region of the targeted locus using the 50 kb ML approach. The difference is perhaps caused by the fact that Fu & Li’s statistics are based on a single category of the frequency spectrum, whereas Tajima’s *D* is based on all categories.

### Power to detect a selective sweep

Often the goal of the investigator is not to estimate the neutrality test statistics in an unbiased manner but instead the goal is to identify regions with extreme values. To investigate our ability to discriminate between regions with selection and neutral regions, we show receiver operating characteristic (ROC) for the different approaches. These are shown in Additional file 6: Figure S6, for different depth, error rates and number of individuals. When the depth is high, all methods perform almost as well as when the genotypes are known without error. But when the depth decreases or the error rates increases, only the full maximum likelihood approach and the EB approach have similar power as the known genotypes.

As expected, the simulations show that our methods have improved power to discriminate between regions evolving neutrally and under positive selection as more samples are added (Additional file 6: Figure S6). The genotype calling methods perform worse when the error rate is increased and the depth is decreased, especially at low depth with a low p-value cutoff, while the ML method for all scenarios performs almost as well as if the true genotypes where known (Additional file 7: Figure S7).

### Variable depth and variable error rates from NGS data

So far, we have used a simple simulation model assuming sequencing depths follow a Poisson distribution and a fixed error rate. To examine the robustness of our conclusions to these assumptions, we made an additional set of simulations using the observed distribution of quality scores and sequencing depths tabulated from BAM files from the 1000 Genomes project (Additional file 8: Figure S8). As before we simulate 100 1 MB regions with and without selection for 25 individuals, and apply our two genotype calling methods and the EB method to the simulated data (Additional file 9: Figure S9). For the genotype calling approaches we only included sites that were likely to be polymorphic with a p-value <10^-6^. We observe results that are highly compatible with the previous results. The EB method is approximately unbiased in both scenarios and have similarly small mean squared error (6e-4, 5e-4). In contrast, the genotype calling methods are biased in both scenarios, and we notice again that the bias depends on whether or not the data is simulated under a neutral or under a selection model. We observe that the MSE for the genotype calling methods are orders of magnitude larger than the EB method, and that the MSE for the GC-mLike model is more than twice as high under the selection scenario as under the neutral scenario, whereas we observe the opposite trend for GC-hwe method.

### Application to real data

**Figure 8:**
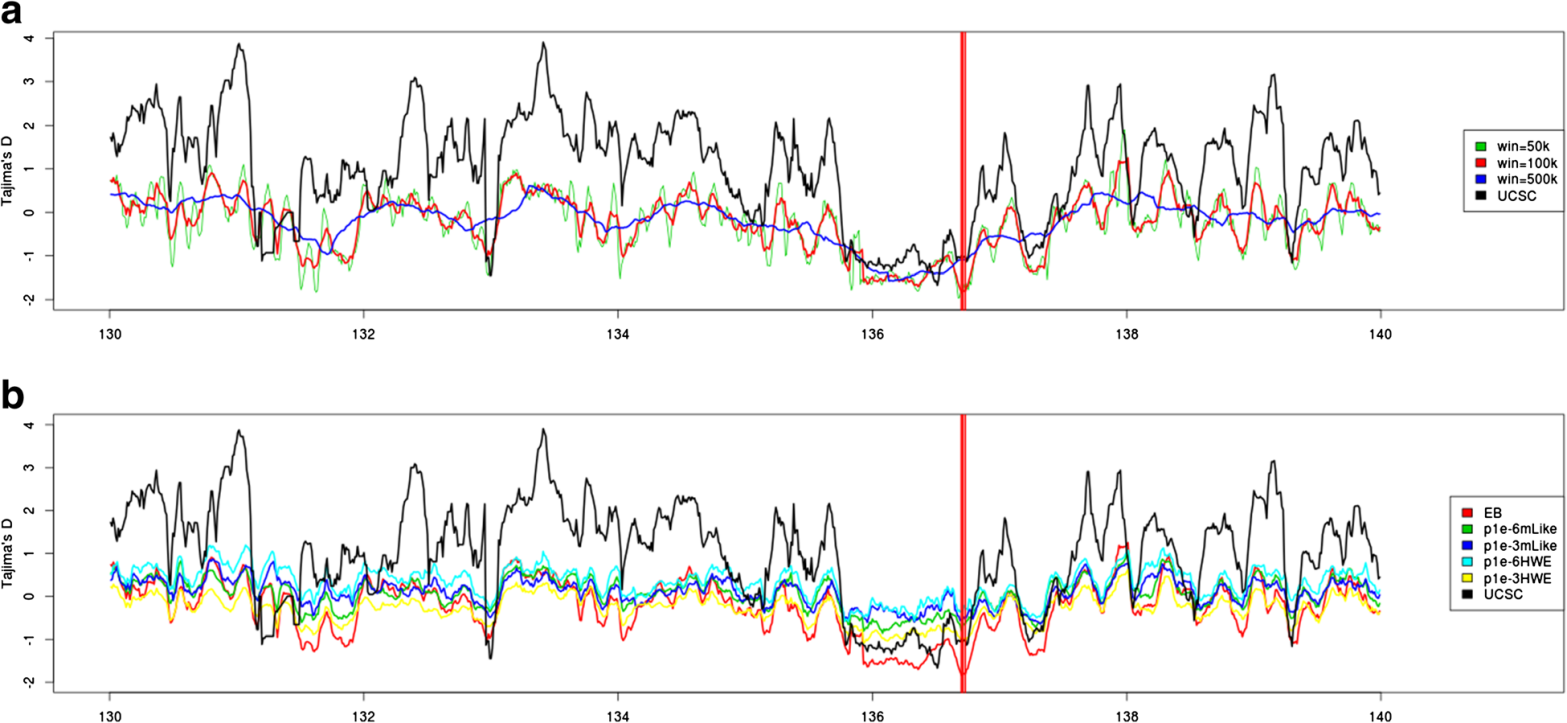
**Applications to real data.** Genomic scans using a sliding window approach of 10 kb. The UCSC Tajima track was downloaded from the UCSC genome browser, and was shifted relatively to LCT gene on the hg19 human assembly. The vertical red line indicates window centers where the EB method (100 kb window) has a Tajima’s *D* below−1.8. Figure **a)** is using our EB method with varying window sizes. Figure **b)** is our EB method together with the genotype calling methods. We tried with varying p-value cutoffs for the genotype calling methods, and are using a window size of 100 kb.

We also applied our methods on real NGS data from the 15 CEU individuals from the 1000 Genomes Project (see method section for details). Bersaglieri et al., 2004 [[5]] found a strong signal of positive selection surrounding the LCT region of chromosome 2 (position 136 Mb). We estimated Tajima’s *D* values in a sliding window across this chromosome, using a prior estimated from a 50 MB region on chromosome 2 (Figure 8). To compare with earlier published results we used the *Tajima track*[43] from the UCSC genome browser [44], which depicts estimated values of Tajima’s *D* from 24 individuals of European descent from the Perlegen genotyping dataset [45]. For our EB method we performed sliding windows analysis with different window sizes (50 kb, 100 kb and 500 kb) all using a fixed step size of 10 kb. We also compared the results for the naïve genotype calling methods using 2 different SNP inclusion cutoff criteria’s (10^-6^, 10^-3^). Notice that the overall estimate of Tajima’s *D* is very positive for the SNP data, most likely due to ascertainment biases [11]. Also notice that the lowest observed value of Tajima’s *D* is the LCT region for the EB approach while there are multiple regions with low Tajima’s *D* values for the GC approaches and the SNP chip data.

## Discussion

We have developed two methods to perform the neutrality tests on NGS data that take the uncertainty of the genotypes into account. Both methods perform better than using called genotypes and in most instances they are approximately unbiased. The full ML method is computationally slow when applied in sliding windows at a genome-wide scale, which is why we also present a fast empirical Bayes method with a prior that is estimated from the data itself, for example the entire genome, or a reasonable subset of the genome. This makes the method computationally feasible for full genomic data of any magnitude and any windows size.

There is not a single obvious way to identify SNP sites and call genotypes from NGS data. Here we have tried different approaches with different cutoffs. Regardless of method and chosen cutoff they all show a large bias in some or all simulated scenarios. This is evident from the raw theta estimates (Table 1), and the actual test statistics (Figure 1). The level of bias varies between the different scenarios, not only for different depths and error rates, but it also depends on whether or not the data set is neutral or affected by selection (Figure 2). The results from this study suggest/confirms it is not unproblematic to perform neutrality tests on genotypes called from low or medium coverage NGS data.

In contrast, both the ML and the EB approach give fairly accurate estimates for all the examined measures. When applying a neutral genome-wide prior for our analysis, we observed only small deviations from the true values of Tajima’s *D* even for very low depth data. When applying the EB approach we did observe a small bias in the regions under selection when the prior was estimated from regions without selection (see Figure 7). However, the bias is always much smaller than the bias of the GC approach. Even though the EB approach can give small biases it can still have an advantage over the full ML approach. When estimating the neutrality test statistics for small windows, we often obtained a few extreme outliers with positive values of Tajima’s *D* for the ML approach (see Figure 6). Since the EB method uses the entire 1 Mb region as prior we do not see a similar problem with extreme positive outliers and the variance of the estimates is smaller overall. If the goal is to identify regions under selection the smaller variance of the EB approach will give fewer regions with extreme values. This is because regions with little data will increase the variance for the full ML approach while they will give results closer to the prior for the EB approach. This problem could also be circumvented by using a sliding window approach with window sizes determined by using a fixed number of SNPs.

We applied the EB method to data from 15 individuals from the 1000 genomes project, and observed trends similar to previous published results for the LCT region in Europeans. For SNP chip data from the same region, we observed a large over-representation of positive Tajima’s *D* values, presumably due to ascertainment biases introduced by the SNP-discovery and selection process from the SNP-chip itself. Similar positive values were not observed using the EB method based on the NGS data. For both the GC and EB methods, we observe negative values around the LCT gene, however the estimates are not very extreme for the GC approach. The EB approach is the only approach for which LCT has the most extreme Tajima’s *D* value.

The main advantage of the approaches presented here, is that they, in expectation, have at most a very weak dependence on sequencing depth. This facilitates the use of genome-wide scans on genomes with varying sequencing depth without introducing biases. The computational framework suggested here, based on the EB approach, provides a robust and computationally fast method for scanning a genome for regions with outlying or extreme frequency spectrum. Such a method should be of great use in years to come when analyzing population genomic data from a variety of different species.

## Conclusion

In this paper we show through simulations that estimating neutrality test statistics using called genotypes can lead to highly biased result. The bias is dependent not only on sequencing depth and error rate but interestingly the bias also depends on whether or not the region is under selection or not. We have developed an empirical Bayes method that can calculate the test statistic fast and efficiently. This method circumvents the problem of SNP discovery, genotype calling and missing data, which is a fundamental problem of NGS data. This is done by working with genotype likelihoods, which contains all relevant information about the uncertainty of the data. Using this approach leads to approximately unbiased estimates in most instances.

## Availability

All methods discussed in this paper are freely available as part of the Analyses of Next generation Sequencing Data (ANGSD) package (http://www.popgen.dk/angsd).

## Competing interest

The authors declare that they have no competing interests.

## Authors’ contributions

RN designed the EB model together with TSK and IM. AA helped with the design of the software package and bug checked early versions of the program. TSK implemented the methods, carried out all analyses and simulations, and drafted the first version of the manuscript with RN. The manuscript has been thoroughly edited by the remaining authors. RN supervised the process. All authors read and approved the final manuscript.

## Supplementary Material

Additional file 1: Figure S1 The effect of genotype calling for low or medium coverage data using Fu & Li’s *D*. The difference between estimated and known Fu&Li’s *D* statistic for three different scenarios with 10 different p-value cutoffs. Each box is estimated on the basis of 100 1 MB regions. The top figure is based on genotypes called using the frequency as prior, and the bottom figure is based on genotypes called using a maximum likelihood approach. Notice that no single best cutoff can be chosen across the three different scenarios. Click here for file

Additional file 2: Figure S2 The effect of genotype calling for low or medium coverage data using Fu & Li’s *F.* The difference between estimated and known Fu&Li’s *F* statistic for three different scenarios with 10 different p-value cutoffs. Each box is estimated on the basis of 100 1 MB regions. The top figure is based on genotypes called using the frequency as prior, and the bottom figure is based on genotypes called using a maximum likelihood approach. Notice that no single best cutoff can be chosen across the three different scenarios. Click here for file

Additional file 3: Figure S3 Difference in Mean Squared Error (MSE) between the full ML and the EB method under a selective sweep. MSE of the estimated Tajima’s *D* (relative to the known expected Tajima’s *D*) is calculated for every 50 kb sub region of the full 1 MB region. The figure is based on 100 1 MB regions. For the EB method we used a prior estimated from the entire 1 MB region on every 50 kb subregion. Click here for file

Additional file 4: Figure S4 Effect of different priors for the EB method using the Fu & Li’s *D.* Left and center plot are boxplots for the difference between our estimate of Fu & Li *D* statistics and the true value, these are based on 100 × 1 Mb regions. Right plot is a 50 kb window plot using the 50 kb ML method along with the EB with neutral and mixed prior. Neutral prior is from a genome-wide prior based on a 100 Mb region, Neu + Sel prior is based on a 200 Mb prior based on 100 Mb selection and 100 Mb neutral. Click here for file

Additional file 5: Figure S5 Effect of different priors for the EB method using the Fu & Li’s *F.* Left and center plot are boxplots for the difference between our estimate of Fu & Li *F* statistics and the true value. Right plot is a 50 kb window plot using the 50 kb method along with the neutral and mixed prior. Neutral prior is from a genome-wide prior based on a 100 Mb region, Neu + Sel prior is based on a 200 Mb prior based on 100 Mb selection and 100 Mb neutral. Click here for file

Additional file 6: Figure S6 Power to detect a selective sweep. ROC curve for scenarios, each plot is based on Tajima’s *D* estimate for 100 × 1 Mb regions with selection and 100 1 MB regions without selection. For each scenario we have our EB method along with our two genotype calling methods (all GC methods is using p-value of 10^-6^). Row1 is 25 individuals row2 is 40 individuals. Column1 is 8× 1% error rate, Column2-4 is all 4×, but with varying error rates 1%,0.5% and 0.1%. Click here for file

Additional file 7: Figure S7 ROC curve for low coverage dataset. ROC curve for a 2×0.5% error rate. The LRT criteria is 10^-6^. This plot is based on 200 1 Mb regions with selection, and 200 1 Mb neutral regions. Click here for file

Additional file 8: Figure S8 Distribution of quality scores and sequencing depth for a BAM file. The left panel shows the quality score distribution and right panel shows the depth distribution, tabulated for chr1 from a BAM file from the 1000 Genomes Project. The mean quality score value was approximately 28 which corresponds to an average error rate of 0.15%. The data covered approximately 87% of the genome, had an average sequencing depth of 4.8, and contained 8,908 sites with a sequencing depth above 100. The right panel only shows the first 30 observations. Click here for file

Additional file 9: Figure S9 Using observed qscore and depth distributions. Boxplots of the difference between the estimate of Tajima’s *D* and the known value for 100 1 MB regions with our EB method and the two genotype calling methods. The quality score and depth distributions used for the genotype likelihood calculations are based on the results depicted in Figure S8. For the genotype calling methods we used a cut-off for the p-value of the LRT test of 10^-6^. Click here for file
