# Supplementary figures and images for: Calculation of Tajima’s D and other neutrality test statistics from low depth next-generation sequencing data
Source: BMC Bioinformatics. 2013 Oct 2;14:289. doi: 10.1186/1471-2105-14-289 (PMC4015034; doi:10.1186/1471-2105-14-289)

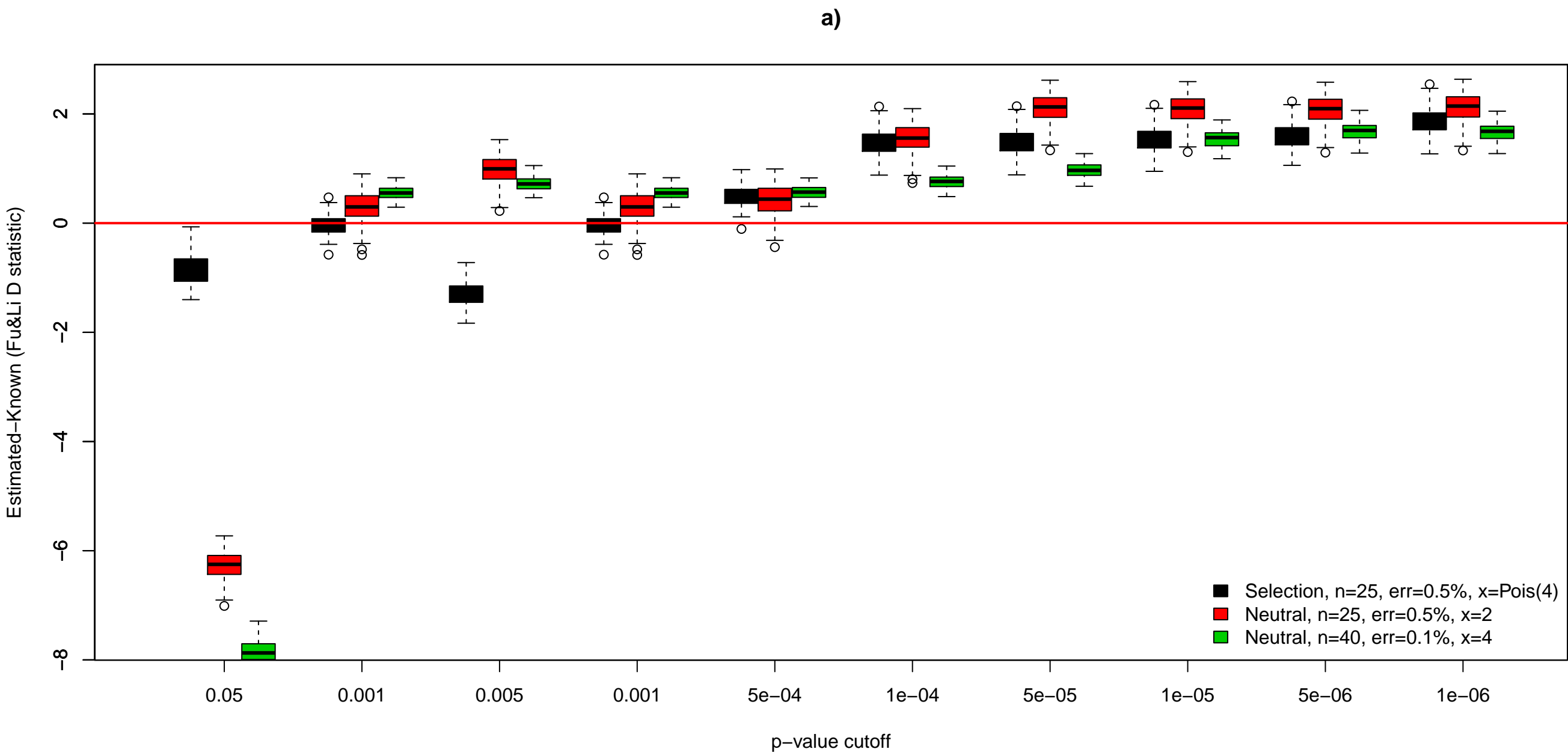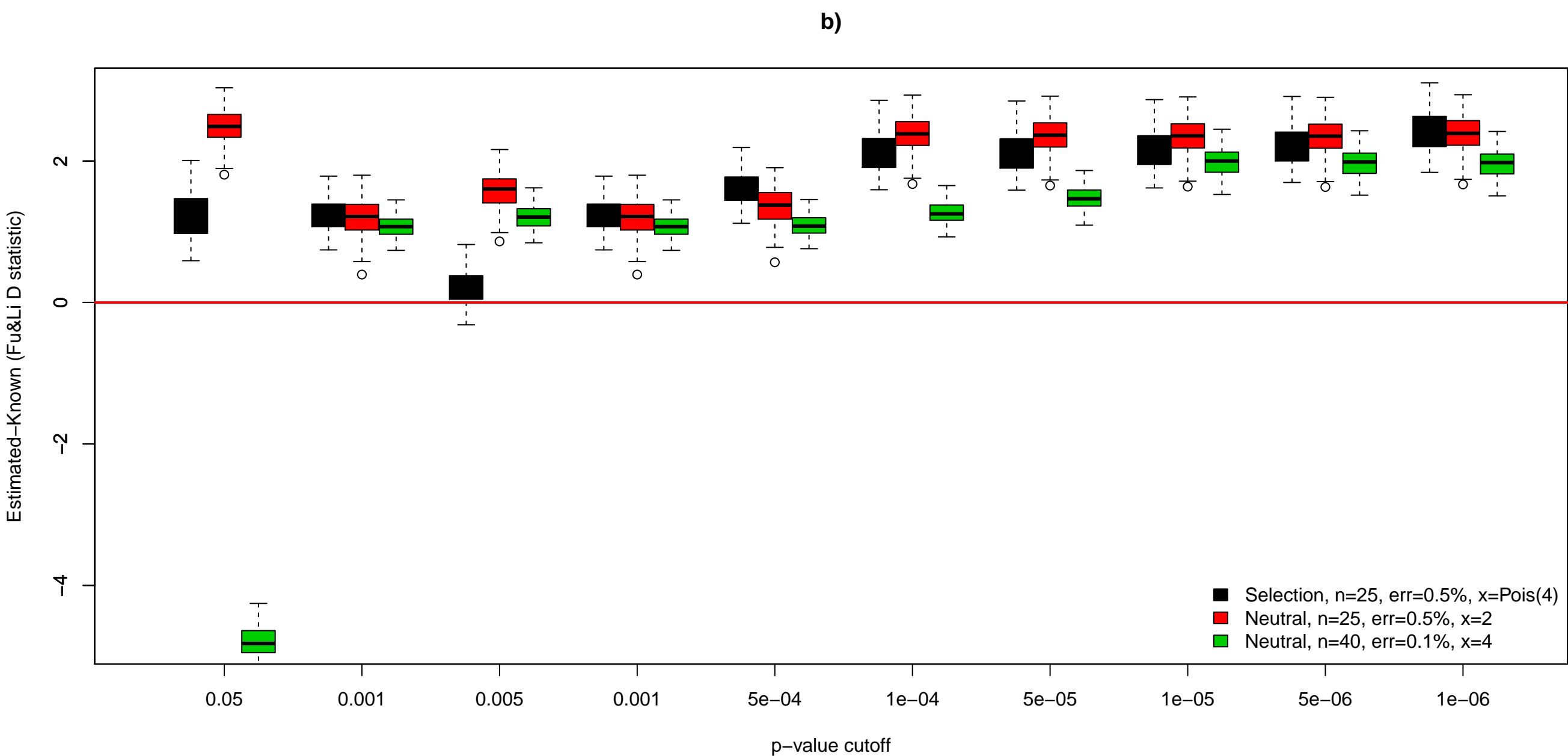

Supplement: Additional file 1: Figure S1 — The effect of genotype calling for low or medium coverage data using Fu & Li’s D. The difference between estimated and known Fu&Li’s D statistic for three different scenarios with 10 different p-value cutoffs. Each box is estimated on the basis of 100 1 MB regions. The top figure is based on genotypes called using the frequency as prior, and the bottom figure is based on genotypes called using a maximum likelihood approach. Notice that no single best cutoff can be chosen across the three different scenarios. [file 1471-2105-14-289-S1.pdf]

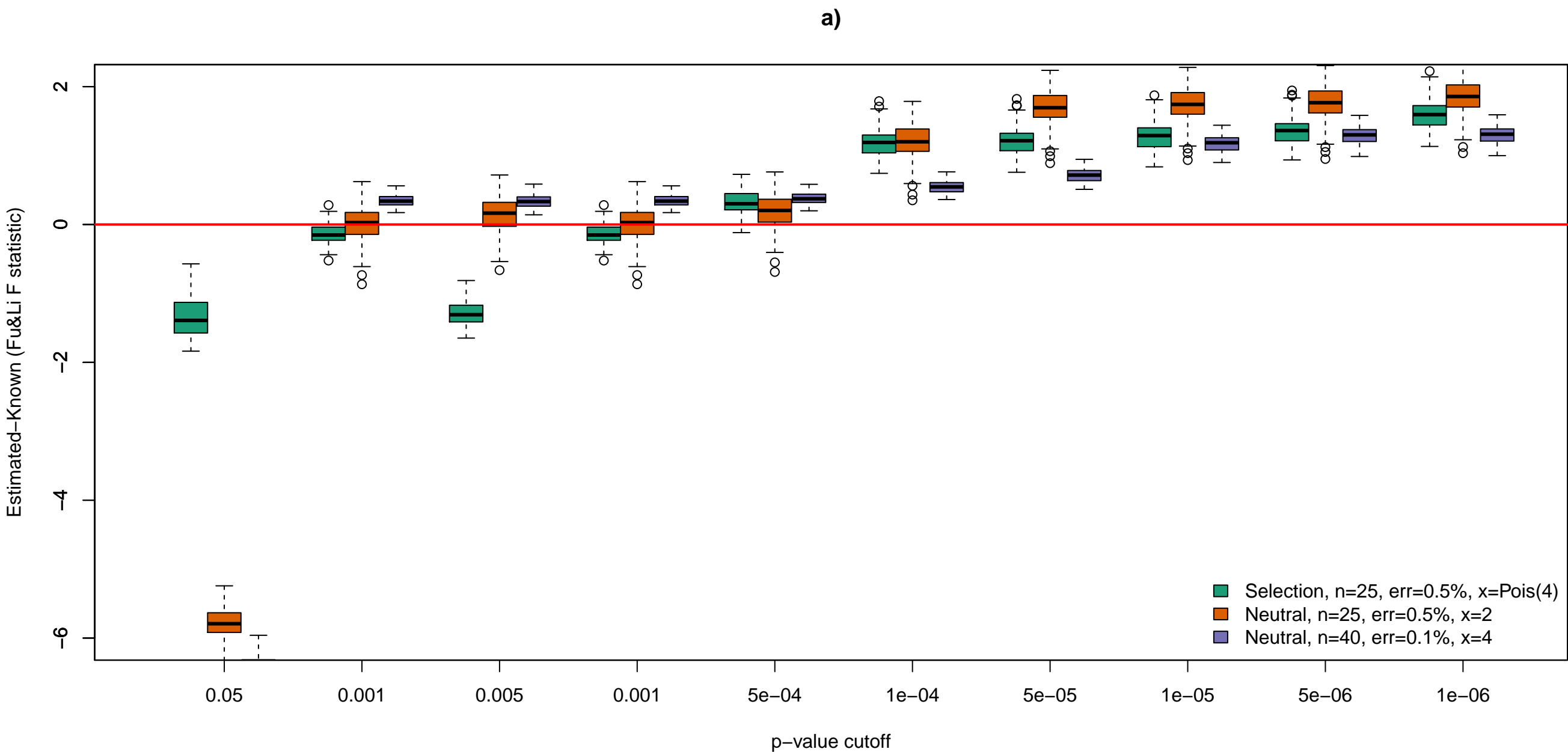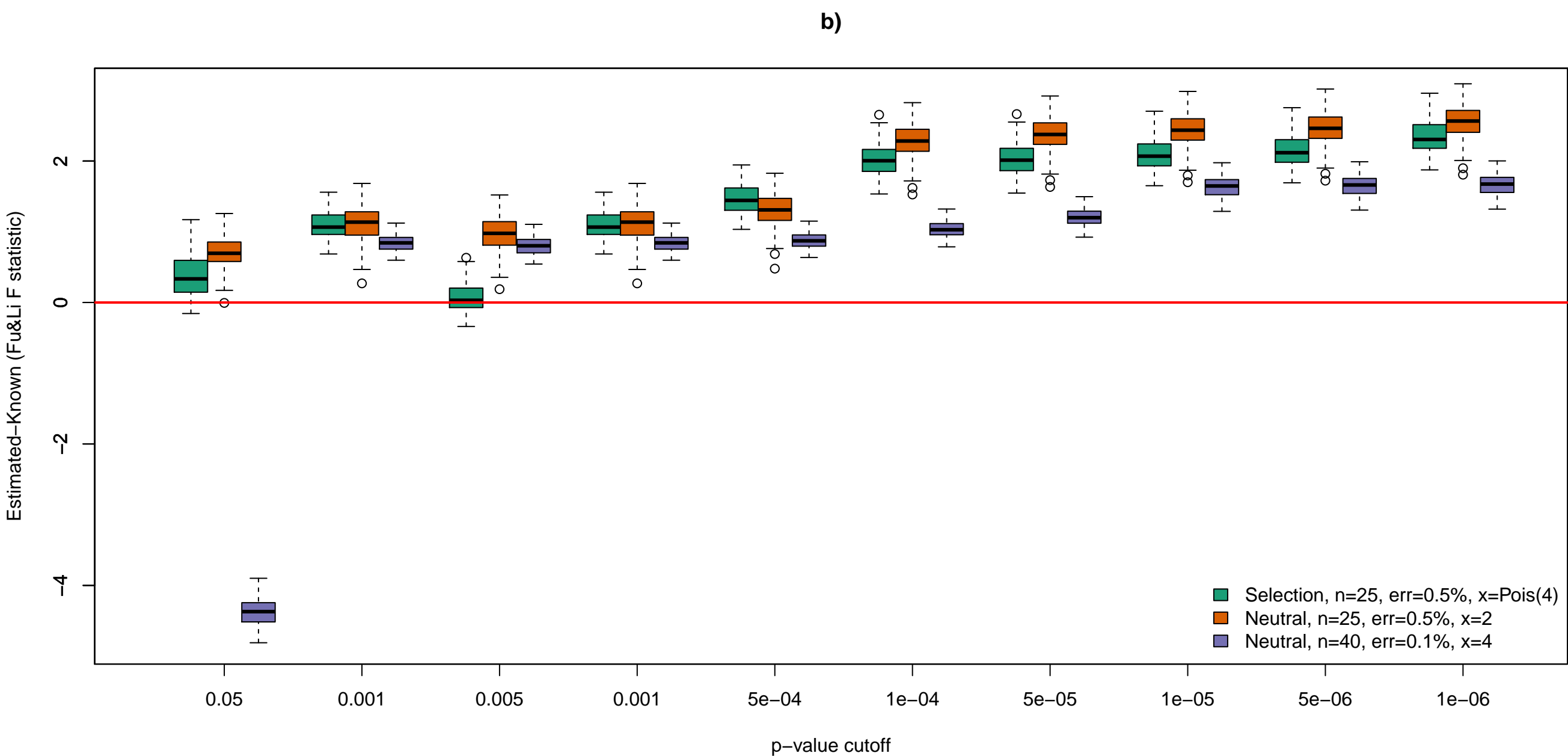

Supplement: Additional file 2: Figure S2 — The effect of genotype calling for low or medium coverage data using Fu & Li’s F. The difference between estimated and known Fu&Li’s F statistic for three different scenarios with 10 different p-value cutoffs. Each box is estimated on the basis of 100 1 MB regions. The top figure is based on genotypes called using the frequency as prior, and the bottom figure is based on genotypes called using a maximum likelihood approach. Notice that no single best cutoff can be chosen across the three different scenarios. [file 1471-2105-14-289-S2.pdf]

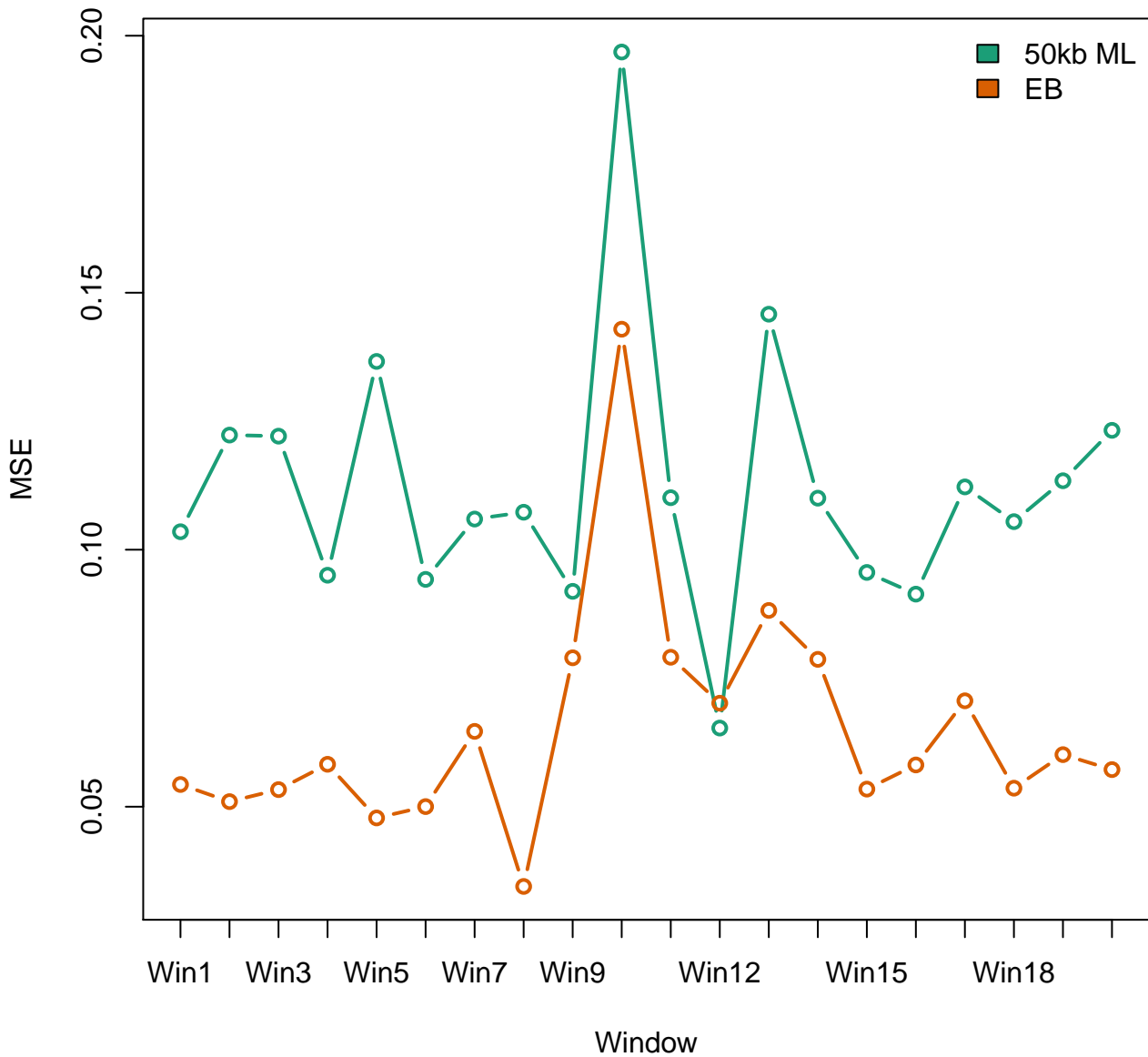

Supplement: Additional file 3: Figure S3 — Difference in Mean Squared Error (MSE) between the full ML and the EB method under a selective sweep. MSE of the estimated Tajima’s D (relative to the known expected Tajima’s D) is calculated for every 50 kb sub region of the full 1 MB region. The figure is based on 100 1 MB regions. For the EB method we used a prior estimated from the entire 1 MB region on every 50 kb subregion. [file 1471-2105-14-289-S3.pdf]

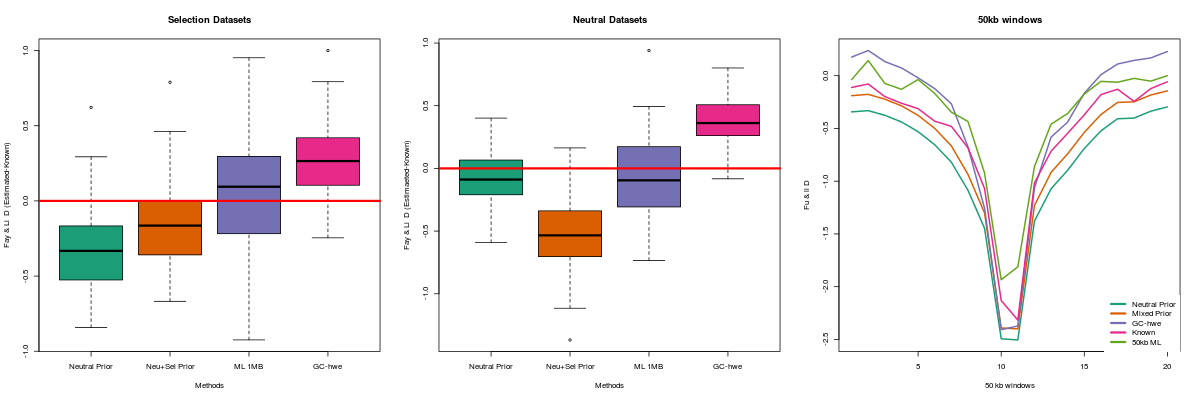

Supplement: Additional file 4: Figure S4 — Effect of different priors for the EB method using the Fu & Li’s D. Left and center plot are boxplots for the difference between our estimate of Fu & Li D statistics and the true value, these are based on 100 × 1 Mb regions. Right plot is a 50 kb window plot using the 50 kb ML method along with the EB with neutral and mixed prior. Neutral prior is from a genome-wide prior based on a 100 Mb region, Neu + Sel prior is based on a 200 Mb prior based on 100 Mb selection and 100 Mb neutral. [file 1471-2105-14-289-S4.tiff]

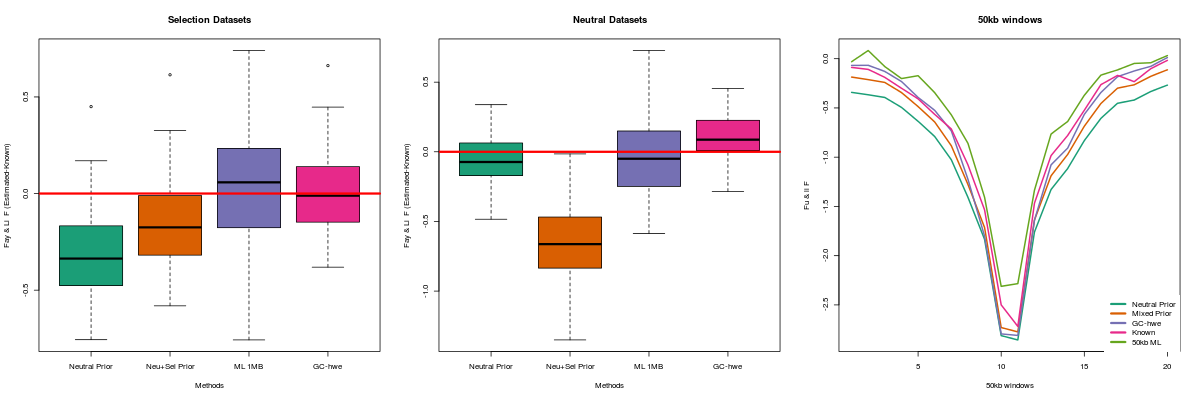

Supplement: Additional file 5: Figure S5 — Effect of different priors for the EB method using the Fu & Li’s F. Left and center plot are boxplots for the difference between our estimate of Fu & Li F statistics and the true value. Right plot is a 50 kb window plot using the 50 kb method along with the neutral and mixed prior. Neutral prior is from a genome-wide prior based on a 100 Mb region, Neu + Sel prior is based on a 200 Mb prior based on 100 Mb selection and 100 Mb neutral. [file 1471-2105-14-289-S5.tiff]

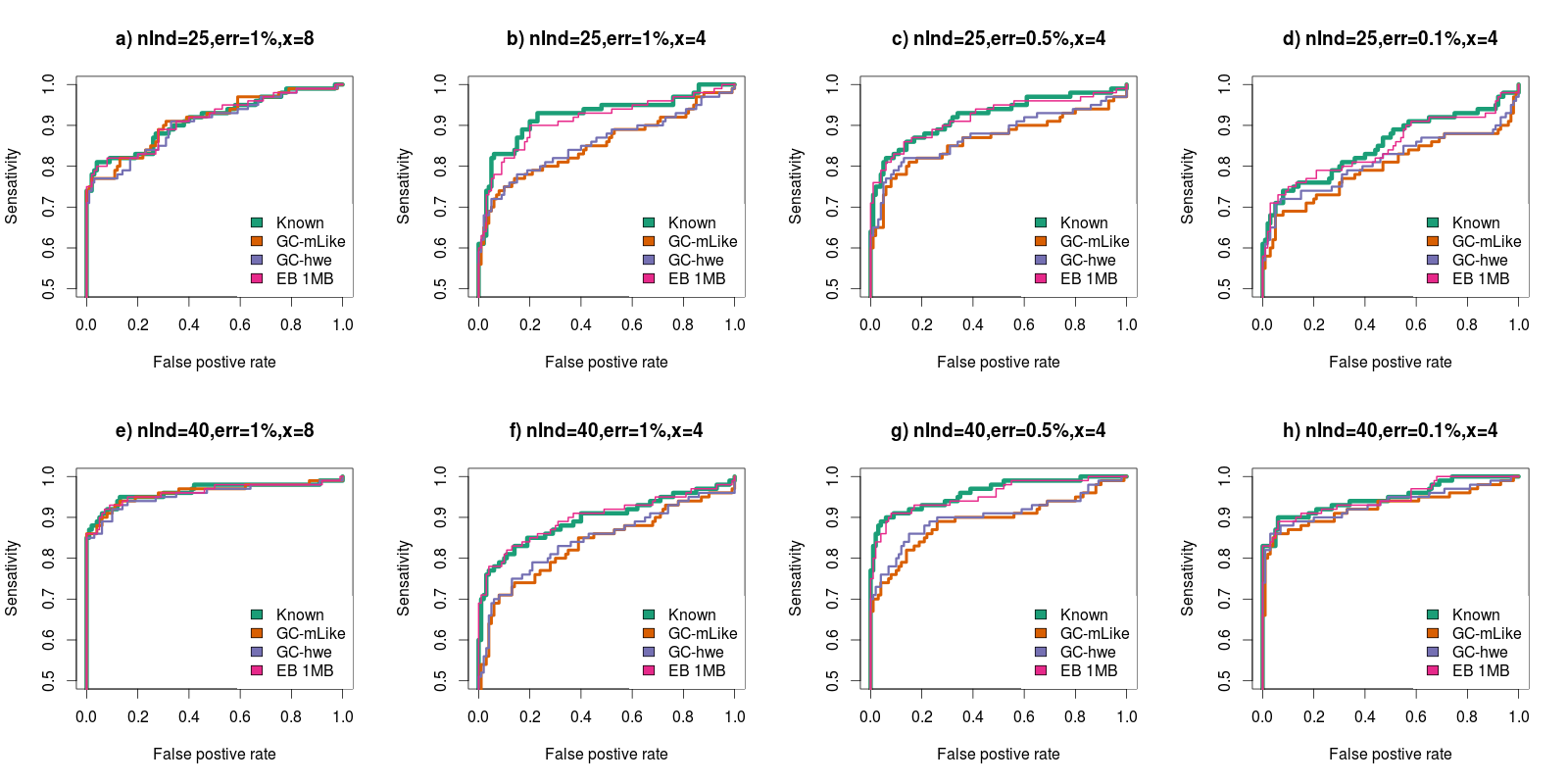

Supplement: Additional file 6: Figure S6 — Power to detect a selective sweep. ROC curve for scenarios, each plot is based on Tajima’s D estimate for 100 × 1 Mb regions with selection and 100 1 MB regions without selection. For each scenario we have our EB method along with our two genotype calling methods (all GC methods is using p-value of 10-6). Row1 is 25 individuals row2 is 40 individuals. Column1 is 8× 1% error rate, Column2-4 is all 4×, but with varying error rates 1%,0.5% and 0.1%. [file 1471-2105-14-289-S6.tiff]

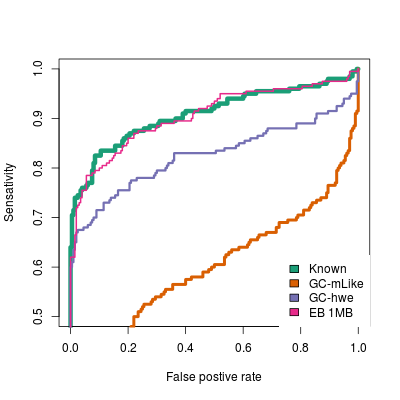

Supplement: Additional file 7: Figure S7 — ROC curve for low coverage dataset. ROC curve for a 2×0.5% error rate. The LRT criteria is 10-6. This plot is based on 200 1 Mb regions with selection, and 200 1 Mb neutral regions. [file 1471-2105-14-289-S7.tiff]

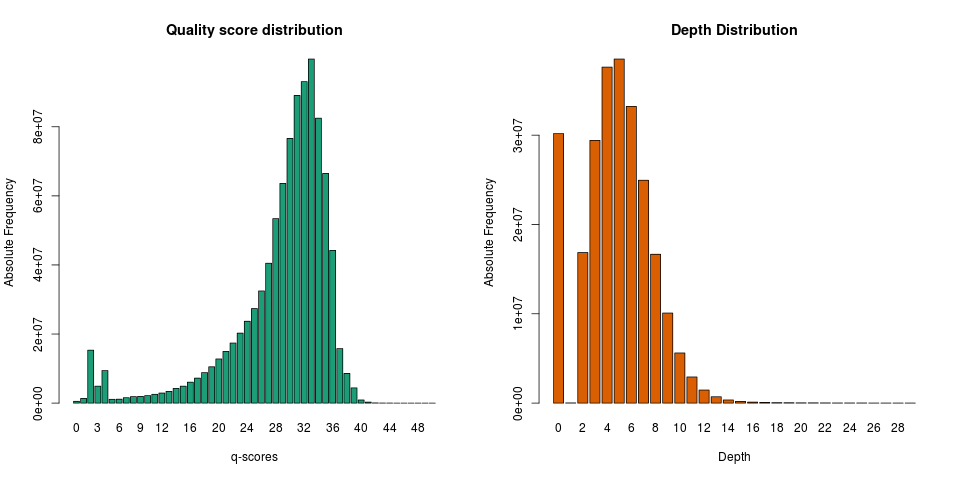

Supplement: Additional file 8: Figure S8 — Distribution of quality scores and sequencing depth for a BAM file. The left panel shows the quality score distribution and right panel shows the depth distribution, tabulated for chr1 from a BAM file from the 1000 Genomes Project. The mean quality score value was approximately 28 which corresponds to an average error rate of 0.15%. The data covered approximately 87% of the genome, had an average sequencing depth of 4.8, and contained 8,908 sites with a sequencing depth above 100. The right panel only shows the first 30 observations. [file 1471-2105-14-289-S8.tiff]

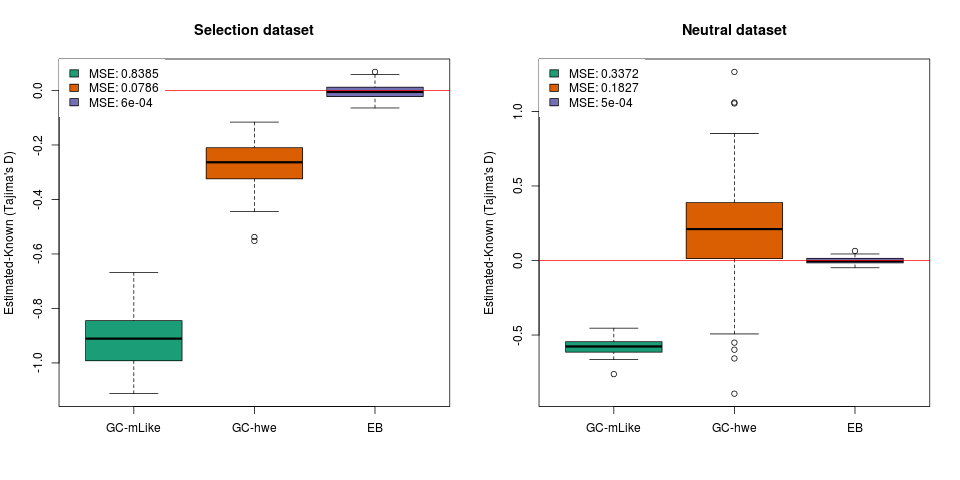

Supplement: Additional file 9: Figure S9 — Using observed qscore and depth distributions. Boxplots of the difference between the estimate of Tajima’s D and the known value for 100 1 MB regions with our EB method and the two genotype calling methods. The quality score and depth distributions used for the genotype likelihood calculations are based on the results depicted in Figure S8. For the genotype calling methods we used a cut-off for the p-value of the LRT test of 10-6. [file 1471-2105-14-289-S9.tiff]
